# CAttSleepNet: Automatic End-to-End Sleep Staging Using Attention-Based Deep Neural Networks on Single-Channel EEG

**DOI:** 10.3390/ijerph19095199

**Published:** 2022-04-25

**Authors:** Tingting Li, Bofeng Zhang, Hehe Lv, Shengxiang Hu, Zhikang Xu, Yierxiati Tuergong

**Affiliations:** 1 School of Computer Engineering and Science, Shanghai University, Shanghai 200444, China; gogogoit@shu.edu.cn (T.L.); hhlv@shu.edu.cn (H.L.); shengxianghu@shu.edu.cn (S.H.); xuzhikangnba@shu.edu.cn (Z.X.); 2 School of Computer and Communication Engineering, Shanghai Polytechnic University, Shanghai 201209, China; 3 School of Computer Science and Technology, Kashi University, Kashi 844008, China; erxat@ksu.edu.cn

**Keywords:** sleep staging, convolutional neural network, attention mechanism, bidirectional long short-term memory, EEG

## Abstract

Accurate sleep staging results can be used to measure sleep quality, providing a reliable basis for the prevention and diagnosis of sleep-related diseases. The key to sleep staging is the feature representation of EEG signals. Existing approaches rarely consider local features in feature extraction, and fail to distinguish the importance of critical and non-critical local features. We propose an innovative model for automatic sleep staging with single-channel EEG, named CAttSleepNet. We add an attention module to the convolutional neural network (CNN) that can learn the weights of local sequences of EEG signals by exploiting intra-epoch contextual information. Then, a two-layer bidirectional-Long Short-Term Memory (Bi-LSTM) is used to encode the global correlations of successive epochs. Therefore, the feature representations of EEG signals are enhanced by both local and global context correlation. Experimental results achieved on two real-world sleep datasets indicate that the CAttSleepNet model outperforms existing models. Moreover, ablation experiments demonstrate the validity of our proposed attention module.

## 1. Introduction

As an important physiological activity, high-quality sleep can effectively restore people’s physical and mental strength, while long-term sleep deprivation or disorder can seriously affect physical and emotional health. It has been shown that certain diseases, such as Parkinson’s disease and Alzheimer’s disease, are strongly associated with sleep disorders or abnormalities [1,2]. Therefore, it is important to improve sleep quality and prevent diseases caused by sleep disorders through a detailed scoring of sleep stages. In the process of sleep staging, sleep experts divide the polysomnography (PSG) into 30 s (30-s) epochs and mark the corresponding sleep stages of each epoch according to the Rechtschaffen and Kales (R&K) [3] and American Academy of Sleep Medicine (AASM) [4] guidelines. Sleep specialists usually label an epoch by analyzing contextual information to find important sleep-related events, such as LAMF and k-complex. However, artificial sleep staging is time-consuming and complex, and the sleep staging results produced by different sleep experts sometimes vary.

Recently, a growing number of researchers have tried to apply artificial intelligence techniques, such as machine learning and deep learning, to solve the issue of sleep staging. Machine learning-based methods usually choose appropriate features from physiological signals (i.e., EEG, EOG, and EMG) [5,6,7,8]. Then, the feature selection algorithm is used to select more representative signal features. Finally, the classifier categorizes sleep stages according to the selected features. Although these approaches have led to some achievements, they still demonstrate some problems. For instance, selecting the most discriminative sleep features requires researchers to have professional experience in the area of sleep medicine, which can be very challenging to obtain. For another, traditional machine learning algorithms are poor at modeling temporal physiological signals.

Deep learning has achieved prominence in various fields (i.e., medical imaging [9,10], emotion recognition [11,12], and bio-signal processing [13]) and provides a novel approach to sleep staging. Some researchers have used convolutional neural networks (CNNs) to construct sleep staging models [14,15,16]. These methods can improve sleep staging performance to some extent. However, traditional CNNs only focus on information within the current receptive field, ignoring context-related information. Therefore, they can easily interfere with non-key features and cannot accurately obtain key local features. Some researchers have gradually come to focus on recurrent neural networks (RNNs) [17,18,19,20,21]. Most of the literature on this subject only considers the contextual association between sleep stages from the global sequence, and only a few works have focused on the local sequence as well. Seo et al. [19] proposed a model called IITNet, which performs feature learning by considering contextual information within and between epochs. Although IITNet has achieved excellent performance, it fails to discriminate the weight of local features.

Feature extraction often plays a decisive role in the results of sleep staging. To obtain more typical feature representations for high-accuracy automatic sleep staging, we propose a novel model named CAttSleepNet. First, local sequences within a 30-s epoch are fed into the conventional CNN module to learn the local sequence features. Second, we feed longer local sequences within a 30-s epoch that are centered on the sequence input to the CNN to the designed attention module. In this way, our proposed attention module can calculate the weights of local sequence features obtained by the CNN network by mining the contextual associations of local sequences. Finally, a two-layer Bi-LSTM is used to encode the global representation of different epochs. Accordingly, our model can more comprehensively capture temporal contextual information and learn the importance of local sequence features. Our main contributions can be summarized as follows.

1. To obtain more discriminative feature representation, our model makes full use of temporal contextual correlation at both local and global levels to achieve high-precision automatic sleep staging on single-channel EEG.
2. To solve the problem whereby traditional CNNs cannot distinguish feature importance due to their limited receptive fields, we add an attention module, which learns the weights of local features by mining the contextual relations of local sequences.
3. Our proposed model is evaluated on the public data sets sleep-edfx-2013 and sleep-edfx-2018. The experimental results show that CAttSleepNet outperforms the existing state-of-the-art methods.

The remainder of the paper is organized as follows. In Section 2, related works on sleep staging are introduced. Section 3 describes CAttSleepNet in detail. Section 4 experimentally evaluates CAttSleepNet. Section 5 concludes the paper and points out directions for future work.

## 2. Related Work

### 2.1. Machine Learning-Based Sleep Staging Methods

Many methods have been proposed for sleep staging by machine learning. For example, Li et al. [5] used 30 EEG signal features, including temporal, frequency, and nonlinear features, to train a random forest model for this task. In [6], the Naive Bayes classifier was applied to classify sleep stages on single-channel EEG. Zhu et al. [7] extracted the graph domain features of EEG and then input these features into a support vector machine model. Seifpour et al. [8] fed extracted EEG time-domain features into a support vector machine for sleep staging. Lajnef et al. [22] proposed a multi-class support vector machine (SVM) classification model based on decision trees. The model used hierarchical clustering techniques and extensive time and frequency domain feature extraction to obtain a decision tree or dendrogram. Hassan et al. [23] utilized bootstrap aggregation (Bagging) and complete ensemble empirical pattern decomposition with adaptive noise (CEEMDAN) to perform a sleep staging task. These sleep staging methods usually require specialized sleep medicine knowledge. In addition, machine learning models do not excel at processing temporal signals.

### 2.2. Deep Learning-Based Sleep Staging Methods

Many studies have applied deep learning to sleep staging. The authors of [14,15,16] used convolutional neural networks for sleep staging. The model shown in reference [14] combined the convolution, max-pooling, and dropout layers. This model used one fully connected layer and the softmax classifier to divide the sleep process into five categories. For this purpose, the classification method proposed in Reference [15] used seven convolution layers, one dropout layer, and one fully connected layer. Traditional CNNs perform well in feature extraction, but fail to exploit the temporal context of sleep stages due to their limited receptive fields.

In recent years, an increasing number of specialists have started to use RNNs in their classification models. Michielli et al. [17] designed a new model based on a cascaded RNN structure with long short-term memory (LSTM) blocks to process single-channel EEG signals. Sun et al. [18] proposed a two-stage sleep staging method—namely, feature learning and sleep pattern recognition. The features in this method were fused manually and trained using CNNs. The fused features were then fed to the RNN module. Supratak et al. [20] combined CNNs with Bi-LSTM, using CNNs to extract representative features from each 30-s epoch and Bi-LSTM to consider contextual correlations for consecutive epochs. The experimental results showed that considering the contextual correlation of signals is an effective technique in automatic sleep staging. Similarly, the model proposed in [21] utilized a three-scale CNN architecture to extract features for each 30-s epoch and fused hand-crafted features with extracted features. Finally, the fusion results were fed into the Bi-LSTM network to learn the transition rules between consecutive epochs. However, most of these models do not consider the contextual correlations of local sequences within a 30-s epoch and ignore the differences in the importance of local features.

## 3. CAttSleepNet

**Figure 1:**
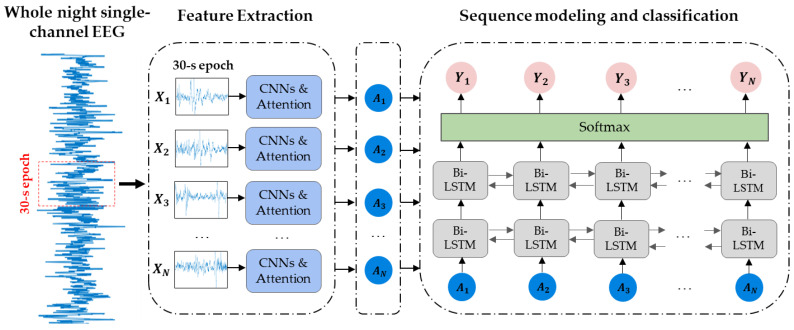
General architecture of CattSleepNet.

Figure 1 shows the overall architecture of the CAttSleepNet model. Firstly, EEG signals, including Fpz-Cz and Pz-Oz channels, are obtained from PSGs. The extracted EEG signals are divided into many 30-s epochs. Next, consecutive epochs are fed into CAttSleepNet to extract their features. After that, we input feature representations of all epochs to a two-layer Bi-LSTM to capture the time dependence of different epochs. The final sleep staging results can be obtained by adding a softmax layer after the two-layer Bi-LSTM.

### 3.1. The Attention-Based CNN for Local Sequence Feature Extraction

According to the AASM manual [4], sleep specialists focus on certain key features when labeling sleep stages, such as low-amplitude mixed frequency (LAMF) and vertex sharp waves in the N1 stage. The variability in the importance of temporal signal features is difficult for standard CNNs to capture, leading models to incorrectly assess key features or even directly ignore them due to their less frequent occurrence. If the CNN is given the ability to pay more attention to high-importance features, the effective extraction of local sequence features and the optimization of the input information of the Bi-LSTM layer can be achieved more easily. Therefore, we designed an attention module to solve this problem.

Attention mechanisms can be categorized as hard attention and soft attention [24]. Hard attention mechanisms filter out the regions of interest as the input, which can help the model to focus on the target object in image processing. However, this method, which directly limits the input content, is not applicable in time series signal classification and prediction. In addition, hard attention mechanisms are more difficult to train and less versatile. In contrast, soft attention mechanisms achieve the purpose of focusing on specific spatial regions or channels by obtaining weights through training learning and then weighting input features on spaces or channels. At the same time, this approach is differentiable in reverse computation, so an end-to-end method can be used to learn the attention network. Based on the above principles, we introduced a soft attention mechanism into the CNN to weight all local sequence features, focus on specific spaces and channels, and achieve the extraction of significant fine-grained features of time series. 

In this work, the local feature extraction consisted of two branches: the standard CNN branch and the attention branch. The standard CNN branch was used for extracting local features. The attention branch was used to compute the corresponding attention scores. Then, the attention score was used to reweight the feature map by element-wise multiplication. This fusion method has been verified to be feasible and effective in various works in the literature [25,26]. Specifically, to obtain richer short sequence features, different scales of inputs were used for the CNN and attention branches. Furthermore, when performing the convolution operation, we set the filter size of the attention branch to be larger than that of the CNN branch. This approach was motivated by previous research [20,27]. Additionally, since the frequency range of sleep stages differs for different time steps [28], we employed different filter kernel sizes to capture the sleep-related frequency band features for different time steps.

**Figure 2:**
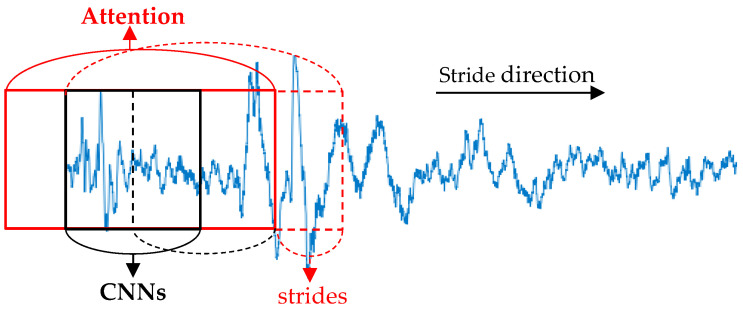
The CNN and attention branches perform feature extraction on the 30-s EEG epoch. The red solid line indicates the input sequence length of attention branch. The black solid line indicates the input sequence length of CNN branch. The dotted line indicates where the two branches will slide forward next.

When given N 30-s EEG epoch ${\text{\{}X}_{1}{,\;X}_{2}{,\;\ldots ,\;X}_{N}\text{\}}$ input to our model, each epoch was input to the CNN and attention module with lengths of 2 s and 4 s. The input sequence of the attention module was centered on the input sequence of the CNN module. In order to prevent the loss of information, two modules slide forward in steps of 1 s. The process is presented in Figure 2. In other words, the 30-s epoch $X_{i}$ can be divided into 29 subsequences of 2 s or 29 subsequences of 4 s, which can be expressed as $X_{i}=\left\{x_{1}^{c}{,\;x}_{2}^{c}{,\ldots ,\;x}_{n}^{c}\right\}=\left\{x_{1}^{a}{,\;x}_{2}^{a}{,\ldots ,\;x}_{n}^{a}\right\}$. $x_{i}^{c}$ represents the i-th short sequence input to the CNN module, and $x_{i}^{a}$ represents the i-th short sequence input to the attention module. This division approach is inspired by the study [29]. We used two modules to extract features from the i-th EEG epoch $X_{i}$, as shown below.

(1) $$h_{i}^{c}=\mathrm{CNN}\left(x_{i}^{c}\right)$$

(2) $$h_{i}^{a}=\mathrm{Attention}\left(x_{i}^{a}\right)$$

(3) $$a_{i\;}{=h}_{i}^{c}\;⊙{\;h}_{i}^{a}$$

where ${\mathrm{CNN}(x}_{i}^{c})$ denotes the operation of the standard CNN module, which can convert the input subsequence $x_{i}^{c}$ into the feature vector $h_{i}^{c}$; ${\mathrm{Attention}(x}_{i}^{a})$ denotes the operation of the attention module, which can convert the input subsequence $x_{i}^{a}$ into the feature vector $h_{i}^{a}$, and ⊙ denotes element-wise multiplication. After the above operations, epoch $X_{i}$ can be represented as a feature vector $A_{I}\;{=\text{\{}a}_{1}{,\;a}_{2}{,\ldots ,\;a}_{n}\text{\}}$. Thereby, the feature vector {$A_{1}$,${\;A}_{2}$, …, $A_{N}$} for N epochs ${\text{\{}X}_{1}{,\;X}_{2}{,\;\ldots ,\;X}_{N}\text{\}}$ can be obtained.

### 3.2. The Two-Layer Bi-LSTM for Global Sequence Modeling

Due to the individual variability of recorded sleep signals, we needed to focus not only on intra-epoch variations, but also on inter-epoch variations to minimize the impact of this variability on the sleep staging task. The simple multiclassification task ignores temporal context information obtained between consecutive sleep epochs. Therefore, we considered the sleep staging problem as a sequential multiclassification task. Specifically, for N EEG epochs ${\text{\{}X}_{1}{,\;X}_{2}{,\ldots ,\;X}_{N}\text{\}}$, CAttSleepNet calculated the output sequence ${\text{\{}Y}_{1}{,\;Y}_{2}{,\ldots ,\;Y}_{N}\text{\}}$ to maximize the conditional probability ${p(X}_{1}{,\;X}_{2}{,\ldots ,\;X}_{N}{|Y}_{1}{,\;Y}_{2}{,\ldots ,\;Y}_{N})$.

In this work, we used a two-layer Bi-LSTM to capture global context information between consecutive sleep epochs. Bi-LSTM was composed of a forward LSTM and a backward LSTM. Therefore, compared to LSTM, Bi-LSTM could utilize the information in both the forward and backward directions. In addition, Bi-LSTM was used to extract coarse-grained features from the significant fine-grained features extracted by the attention-based CNN network. Meanwhile, it prevented the memory loss and gradient dispersion problems caused by the use of excessively long steps. In conclusion, a two-layer Bi-LSTM could capture the temporal dependence of consecutive epochs to achieve coarse and fine-grained feature fusion and fully characterize time-series data.

For the feature representation ${\text{\{}A}_{1}{,A}_{2}{,\ldots ,A}_{N}\text{\}}$ of N 30-s EEG epoch ${\text{\{}X}_{1}{,\;X}_{2}{,\ldots ,\;X}_{N}\text{\}}$, where $A_{i}{=\text{\{}a}_{1}{,a}_{2}{,\ldots ,a}_{n}\text{\}}$, we modeled the global sequence between 30-s epochs, as follows.

(4) $$\vec{H_{t}}\;=\mathrm{LSTM}\left(A_{t},\;\vec{H_{t-1}},\;\vec{C_{t-1}}\right)$$

(5) $$\overset{\leftarrow }{H_{t}}\;=\mathrm{LSTM}\left(A_{t},\;\overset{\leftarrow }{H_{t+1}},\;\overset{\leftarrow }{C_{t+1}}\right)$$

(6) $$O_{t}=\vec{H_{t}}\;\|\;\overset{\leftarrow }{H_{t}}$$

where LSTM(·) denotes the operation of a two-layer LSTM, which can model the feature vector $A_{t}$ from the front and back directions; C and H are the vectors of cells and hidden states; and ‖ is a concatenation operation. Finally, sleep staging results can be obtained by adding a softmax layer after a two-layer Bi-LSTM.

### 3.3. Model Training and Parameter Optimization

**Figure 3:**
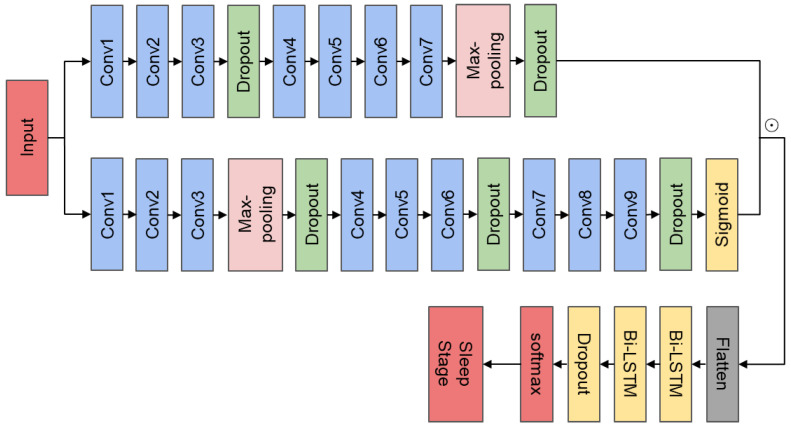
Detailed structure of CAttSleepNet model.

Figure 3 shows the specific structure of CAttSleepNet. The top branch is the CNN module, and the middle is the attention module. Table 1 displays the specific parameters of CAttSleepNet. The CNN branch consisted of seven one-dimensional convolution layers, one max-pooling layer, and two dropout layers. The attention branch contained nine one-dimensional convolutional layers, one max-pooling layers, and three dropout layers. To solve the problem of overfitting during training, we used a dropout layer with the parameter set to 0.5. In particular, the batch normalization and application of corrected linear unit (ReLU) activation were performed for each convolutional layer in two branches. The attention branch restricted feature values extracted through a series of convolution and pooling operations between 0 and 1, through a sigmoid function. The output features of the CNN branch were multiplied element-wise with the output weights of the corresponding attention branch. Specifically, the more important the feature of the CNN branch was, the closer the output weight of the corresponding attention branch was to 1. On the contrary, the less important the feature of the CNN branch was, the closer the output weight of the corresponding attention branch was to 0.

We input five minutes EEG segments—i.e., ten 30-s EEG epochs—into the model without preprocessing for end-to-end automatic sleep staging. The CNN and the corresponding attention branches were slid forward simultaneously in one-second steps. The sliding window size of the CNN block was 2 s, and the sliding window size of the attention block was 4 s. Since the sampling rate of EEG was 100 Hz, per second EEG signal contained 100 data points. After the attention-based CNN module, the vector of shape (1, 256) was obtained. Fused results were flattened and fed to a two-layer Bi-LSTM. The number of hidden units in the Bi-LSTM layer was 64. Eventually, the softmax classifier outputs the most likely sleep stage.

We utilized the Adam optimizer with a learning rate of 0.001 to optimize the model parameters. CAttSleepNet could be trained end-to-end using the back-propagation algorithm. The cross-entropy loss function was employed. The loss function was defined as follows.

(7) $$\mathrm{loss}=-\frac{1}{S}\sum_{k=0}^{K-1}\sum_{i=0}^{S-1}y_{i,k}\log\left(p_{i,k}\right)$$

where K denotes the number of classes; S denotes the total number of samples; and $y_{i,k}$ and $p_{i,k}$ denote the actual label and predicted probability of the i-th sample for class k, respectively. The maximum training epoch was set to 300. Moreover, we performed our experiments on a device with two GPUs (NVIDIA GeForce GTX 1080 Ti) using Python 3.6 and Tensorflow 1.10. For the sleep-edfx-2013 dataset, each training epoch took about 1 min, while the sleep-edfx-2018 dataset needed roughly 4 min.

## 4. Experimental Evaluation

### 4.1. Experiment Datasets and Evaluation Metrics

#### 4.1.1. Experiment Datasets

In this experiment, we used the public Physionet sleep-edf expanded (sleep-edfx) dataset [30,31], including version 1 from 2013 and version 2 from 2018, to evaluate the performance of CAttSleepNet. The sleep-edf dataset was first released in 2002 (version 0), and only contained a small amount of data; thus, it was not used in our study. It was expanded in 2013 and 2018, with sleep-edfx-2013 containing 61 whole-night PSGs, while sleep-edfx-2018 reached 197 whole-night PSGs. Additionally, these sleep signals were derived from two different studies—namely, studies on the impact of age on healthy adults (SC) and the effect of temazepam medication on sleep (ST). We only utilized data from * SC files for this task. Subjects’ EEG (Fpz-Cz and Pz-Oz channels), EOG, EMG, and event markers were recorded in each PSG. The sampling rates of EEG, EOG, and EMG were 100 Hz, 100 Hz, and 1 Hz, respectively. Some PSGs also recorded respiration and body temperature. These PSGs were manually labeled by professionals following the R&K manual [3]. Specifically, each 30-s epoch was labeled with one of the following classes: wakefulness (W), rapid eye movement (REM), MOVEMENT, UNKNOWN, and non-rapid eye movement (NREM, which was further divided into N1, N2, N3, and N4 stages). We combined N3 and N4 into N3 according to the AASM manual [4] and removed the MOVEMENT and UNKNOWN stages. Therefore, sleep stages were classified into five categories: W, REM, N1, N2, and N3. Furthermore, we eliminated some W stages and retained only the 30-min waking periods before and after sleep. This approach was consistent with the study [20]. As shown in Table 2, we performed sleep staging using EEG signals from Fpz-Cz and Pz-Oz channels.

#### 4.1.2. Evaluation Metrics

K-fold cross-validation can improve the learning ability of deep learning models and make them more robust. In this experiment, we applied the k-fold cross-validation method to evaluate the performance of the CAttSleepNet model. To ensure a fairer comparison of the experimental results, the k values in this paper were set to be the same as those studies [20,27,29,32,33,34,35,36,37]—i.e., the k values were taken as 20 and 10 on the sleep-edfx-2013 and sleep-edfx-2018 datasets, respectively. The detailed process was as follows:

1. The sleep-edfx-2013 and sleep-edfx-2018 datasets are shuffled into k equal parts. K was set to 20 and 10, correspondingly.
2. One of the k equal parts was taken as a test set and the rest as a training set.
3. We trained the model and calculated the accuracy on the test set.

Steps 2 and 3 were repeated k times, and then the average of k test results was calculated as the final result.

To more comprehensively assess the behavior of our model in the sleep staging task, we considered the model evaluation both overall and per-category separately. On the one hand, due to the different number of samples for each sleep stage, we used the overall accuracy (ACC), Macro-F1 score (MF1), and Cohen’s Kappa coefficient (K) [38,39] to obtain a more intuitive and realistic portrayal of the overall classification. On the other hand, we calculated precision, recall, and F1-score for each class separately. The calculation formula used for the above indicators was as follows.

(8) $${\mathrm{precision}}_{i}=\frac{{\mathrm{TP}}_{i}}{{\mathrm{TP}}_{i}{+\mathrm{FP}}_{i}}$$

(9) $${\mathrm{recall}}_{i}=\frac{{\mathrm{TP}}_{i}}{{\mathrm{TP}}_{i}{+\mathrm{FN}}_{i}}$$

(10) $${F1}_{i}=\frac{{2\;\times \;\mathrm{precision}}_{i}{\;\times \;\mathrm{recall}}_{i}}{{\mathrm{precision}}_{i}{+\mathrm{recall}}_{i}}$$

(11) $$\mathrm{ACC}=\frac{\sum_{i=1}^{C}{\mathrm{TP}}_{i}}{S}$$

(12) $$\mathrm{MF}1=\frac{1}{C}\sum_{i=1}^{C}{F1}_{i}$$

(13) $$K=\frac{p_{o}-{\;p}_{e}}{1\;-{\;p}_{e}}$$

where ${\mathrm{FP}}_{i}$, ${\mathrm{FN}}_{i}$, and ${\mathrm{TP}}_{i}$ are false positive, false negative, and true positive for the i-th class, respectively; S is the total amount of samples; and C is the number of categories. In this experiment, C is set to 5. $p_{o}$ is the actual agreement rate, while $p_{e}$ is the theoretical agreement rate.

### 4.2. Experimental Results of CAttSleepNet

**Figure 4:**
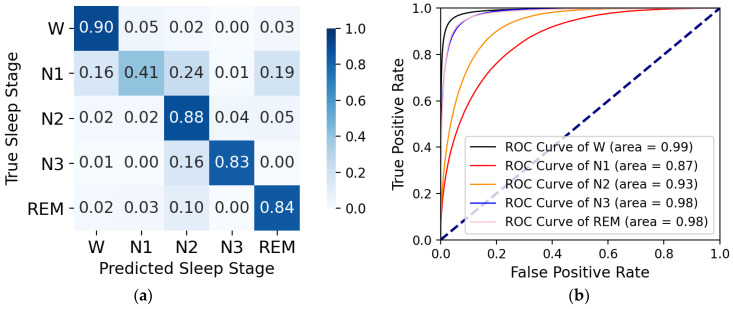
The CAttSleepNet model’s confusion matrix and ROC curve are obtained from the Fpz-Cz channel of the sleep-edfx-2013 dataset. (**a**) Confusion matrix; (**b**) ROC curve.

**Figure 5:**
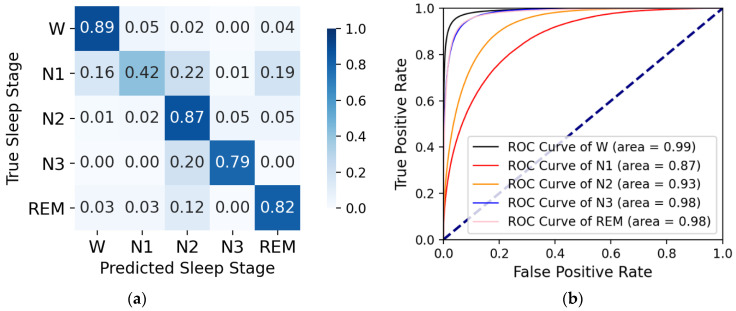
The CAttSleepNet model’s confusion matrix and ROC curve are obtained from the Pz-Oz channel of the sleep-edfx-2013 dataset. (**a**) Confusion matrix; (**b**) ROC curve.

**Figure 6:**
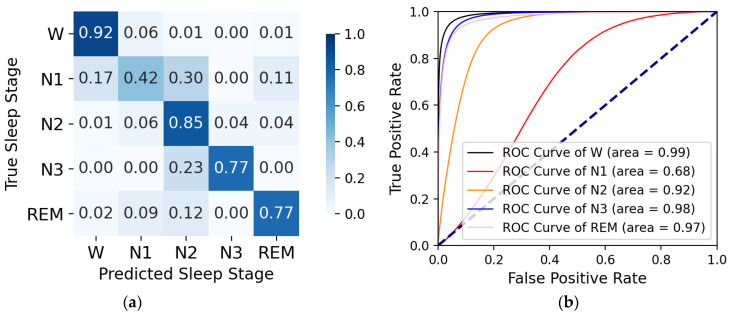
The CAttSleepNet model’s confusion matrix and ROC curve are obtained from the Fpz-Cz channel of the sleep-edfx-2018 dataset. (**a**) Confusion matrix; (**b**) ROC curve.

**Figure 7:**
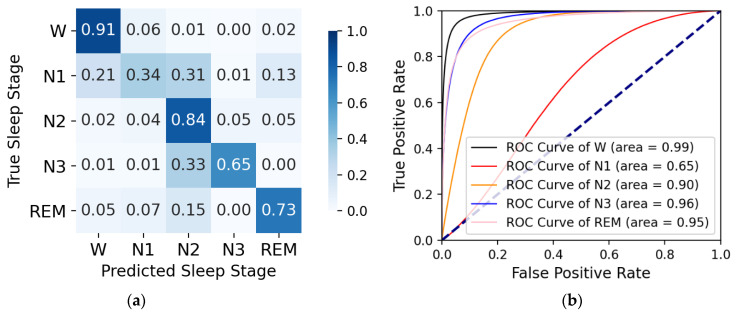
The CAttSleepNet model’s confusion matrix and ROC curve are obtained from the Pz-Oz channel of the sleep-edfx-2018 dataset. (**a**) Confusion matrix; (**b**) ROC curve.

On the sleep-edfx-2013 and sleep-edfx-2018 datasets, we obtained four k-fold cross-validation confusion matrices and corresponding receiver operating characteristic (ROC) curves, as shown in Figure 4, Figure 5, Figure 6, and Figure 7. In the confusion matrices, diagonal positions represent the proportions of correct classification, and other positions indicate proportions misclassified as other classes. The darker the color is, the higher the percentage is. For the sleep-edfx-2013 dataset, except for the N1 stage, the other four classes achieved a high accuracy. The highest classification accuracy was obtained for the W and N2 stages. The classification accuracy of the N1 stage on two channels was 41% and 42%, respectively. N1 stages were mainly misclassified as N2 and REM stages. Due to the small number of samples and few features learned during training, the N1 stage was the most indistinguishable among the five classes. The confusion matrix distribution of the sleep-edfx-2018 dataset was similar to that of the sleep-edfx-2013 dataset. On the other hand, the same ROC curve was obtained, since our model obtained similar classification results for both channels on the sleep-edfx-2013 dataset. The ROC curves on the two channels of sleep-edfx-2018 were vastly different. The CAttSleepNet model had the highest area under curve (AUC) on the sleep-edfx-2013 dataset and the lowest AUC on the Pz-Oz channel of sleep-edfx-2018. This may be due to the poor performance of the CAttSleepNet model on the Pz-Oz channel of sleep-edfx-2018 classification for each sleep stage, especially for the N1 stage.

Moreover, Table 3 also displays the ACC, MF1, and K of the overall classification, precision, recall, and F1-score for each category. It is obvious that our model outperformed the Pz-Oz channel on the Fpz-Cz channel, both overall and per-class. On the sleep-edfx-2013 dataset, the ACC, MF1, and K of the Fpz-Cz channel were 1.56%, 2.12%, and 1.51% higher than those of the Pz-Oz channel, respectively. On the sleep-edfx-2018 dataset, they were 2.8%, 4.06%, and 4.48%, correspondingly. On the other hand, the above indicator values for the sleep-edfx-2013 dataset were significantly higher than those for the sleep-edfx-2018 dataset.

### 4.3. Comparison with State-of-the-Art Methods

In this section, we compare the performance of our model with that of existing models [20,27,29,32,33,34,35,36,37]. Reference [32] used convolutional neural networks to automatically score sleep stages on the Fpz-Cz channel of EEG without using prior knowledge. Reference [33] designed a sleep staging model with stacked sparse autoencoders. Supratak et al. [20] proposed the DeepsleepNet model for sleep staging. The literature [29] used an approach combining attention mechanisms and bidirectional recurrent neural networks. Meanwhile, the literature [34] utilized a 1-max pooling CNN and time-frequency image features for automatic sleep staging. Additionally, a CNN framework for joint classification and prediction was proposed in the study [35]. Zhu et al. [36] proposed an automatic sleep staging method based on the attention mechanism and convolutional neural networks. Yang et al. [27] designed the 1D-CNN-HMM model, which combines the hidden Markov model (HMM) and one-dimensional convolutional neural network (1D-CNN). A new model for automatic sleep staging called SleepEEGNet, was proposed in the work of [37].

The results of our method compared with those of other methods are shown in Table 4. The validation of our experiments was consistent with these methods—i.e., using 20-fold cross-validation and 10-fold cross-validation on the sleep-edfx-2013 and sleep-edfx-2018 datasets, separately. On the sleep-edfx-2013 dataset, our model achieved the best results in terms of the overall metrics (ACC, MF1, and K) for both channels. That is, the ACC, MF1, and K on the Fpz-Cz channel were 84.1%, 78.2%, and 78%, respectively, while the ACC, MF1, and K on the Pz-Oz channel were 82.58%, 76.69%, and 76%, respectively. Second, the CAttSleepNet model achieved the highest F1-score for each class on the Pz-Oz channel, and the highest F1-score for the N1 and N2 stages on the Fpz-Cz channel. It is more difficult to achieve satisfactory classification results in N1 stages because of the smaller sample and more similar modality to the N2 stages, as is verified by experimental results recorded in the literature [20,27,29,32,33,34,35,36,37]. Without dealing with class imbalance, our model still outperformed these methods in the N1 stage. There have been few studies on the sleep-edfx-2018 dataset so far, and we only compared our results with those in the literature [37]. Our model outperformed that in the study of [37] in terms of overall classification metrics, performing satisfactorily in each class. In addition, we can see that the models of [36,37] achieved better or similar classification results compared to our model in the W, N1, and REM stages; this may be due to the different contributions of different classifiers to the different categories. Moreover, in the literature [36,37], there was a greater number of samples in the W stages, which may also have affected the actual classification results. In summary, CAttSleepNet achieved a highly competitive performance compared to that of other well-established sleep staging models.

### 4.4. Ablation Experiment

**Figure 8:**
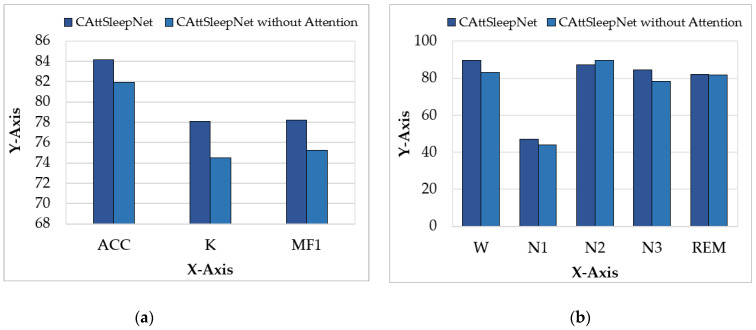
Visual comparison of the two models. (**a**) Comparison of overall indicators; (**b**) comparison of per-class F1-score.

To verify the validity of the attention approach proposed in this paper, we conducted ablation experiments. Without changing the model parameters, we compare the performance of CAttSleepNet with and without attention in the Fpz-Cz channel of the sleep-edfx-2013 dataset. Additionally, both models used the same optimizer, loss function, and experimental environment. We calculated the ACC, MF1, and K, and per-class F1-score for the two models separately. Table 5 shows the experimental results obtained. The model with the attention branch outperformed the model without the attention branch. Specifically, ACC improved by 2.19%, K increased by 3.59%, and MF1 was enhanced by 2.94%. The visualization comparison results are shown in Figure 8. It can be seen from the figure that with the attention branch, CattSleepNet achieved better or similar classification results for each sleep stage. With the attention branch, the performance of CAttSleepNet was more consistent with the actual classification results. Therefore, the values of K and MF1 also increased significantly.

## 5. Conclusions

In this paper, we proposed a deep learning model named CAttSleepNet for automatic end-to-end sleep staging based on raw single-channel EEG. First, the attention-based CNN architecture could calculate the weights of local features by mining contextual associations; thus, it could differentiate the importance of key and non-key local features. Second, a two-layer Bi-LSTM was applied to globally model consecutive epochs, enabling end-to-end automatic sleep staging by exploiting the transition rules between sleep stages. The experimental results obtained on the sleep-edfx-2013 and sleep-edfx-2018 datasets demonstrate that our model achieved a better performance than the existing models. Furthermore, ablation experiments proved the effectiveness of our proposed attention module for use in sleep staging. Although our model achieved a promising performance, it still had some shortcomings. In the future, we hope to fuse information from multiple modalities, such as EOG and EMG, to enhance the performance of CAttSleepNet.

## Figures and Tables

**Table 1:** Detailed parameters of CAttSleepNet model.

Branch	Layer Type	Number of Filters	Kernel Size	Region Size	Stride	Output Shape
**Input**	**-**	**-**	**-**	**-**	**-**	(200, 1)
**The CNN**	Conv1	64	1 × 5	-	3	(67, 64)
	Conv2	64	1 × 5	-	3	(23, 64)
	Conv3	64	1 × 5	-	3	(8, 64)
	Conv4	128	1 × 3	-	2	(4, 128)
	Conv5	128	1 × 3	-	2	(2, 128)
	Conv6	128	1 × 3	-	1	(2, 128)
	Conv7	256	1 × 3	-	1	(2, 256)
	Max-pooling	-	-	1 × 2	1	(1, 256)
**Input**	-	-	-	-	-	(400, 1)
**The Attention**	Conv1	64	1 × 7	-	3	(134, 64)
	Conv2	64	1 × 7	-	3	(45, 64)
	Conv3	64	1 × 7	-	3	(15, 64)
	Max-pooling	-	-	1 × 2	2	(8, 64)
	Conv4	128	1 × 5	-	2	(4, 128)
	Conv5	128	1 × 5	-	2	(2, 128)
	Conv6	128	1 × 5	-	2	(1, 128)
	Conv7	256	1 × 3	-	1	(1, 256)
	Conv8	256	1 × 3	-	1	(1, 256)
	Conv9	256	1 × 3	-	1	(1, 256)

**Table 2:** Detailed distribution of sleep stages in sleep-edfx-2013 and sleep-edfx-2018 datasets.

Stage	Sleep-Edfx-2013			Sleep-Edfx-2018		
	Training Set	Test Set	Total	Training Set	Test Set	Total
W	7734	292	8026	55,697	10,023	65,720
N1	2666	138	2804	19,207	2315	21,522
N2	16,805	994	17,799	89,789	6343	96,132
N3	5449	254	5703	11,879	1160	13,039
REM	7295	422	7717	23,452	2383	25,835
**Total**	**39,949**	**2100**	**42,049**	**200,024**	**22,224**	**222,248**

**Table 3:** Evaluation indicators for the overall and each category are obtained from two datasets.

	Sleep-Edfx-2013						Sleep-Edfx-2018					
	EEG Fpz-Cz (%)			EEG Pz-Oz (%)			EEG Fpz-Cz (%)			EEG Pz-Oz (%)		
	Pre	Rec	F1	Pre	Rec	F1	Pre	Rec	F1	Pre	Rec	F1
W	88.86	90.28	89.56	89.56	89.44	89.50	92.56	91.67	92.12	89.24	90.67	89.95
N1	55.59	40.87	47.11	51.98	41.93	46.42	46.10	41.61	43.74	45.79	34.33	39.24
N2	86.24	88.21	87.22	84.63	87.10	85.84	81.71	84.90	83.28	78.52	83.79	81.07
N3	86.57	83.47	84.50	82.55	79.38	80.94	77.25	76.66	76.96	71.18	65.20	68.06
REM	80.05	84.30	82.12	79.45	82.09	80.75	76.51	76.89	76.70	71.01	73.16	72.07
Overall Indicators	**ACC**	**K**	**MF1**	**ACC**	**K**	**MF1**	**ACC**	**K**	**MF1**	**ACC**	**K**	**MF1**
	84.14	78.09	78.20	82.58	75.97	76.69	80.81	73.51	74.56	78.01	69.45	70.08

Note: Pre = precision, Rec = recall, F1 = F1-score.

**Table 4:** Comparison among CAttSleepNet and other models.

Approach	Overall Performance (%)			Per-Class F1-Score (%)				
	ACC	MF1	K	W	N1	N2	N3	REM
Dataset: Sleep-Edfx-2013 EEG Channel: Fpz-Cz								
Tsinalis et al. [32]	74.8	69.8	-	65.4	43.7	80.6	84.9	74.5
Tsinalis et al. [33]	78.9	73.7	-	71.6	47.0	84.6	84.0	81.4
Supratak et al. [20]	82.0	76.9	0.76	84.7	46.6	85.9	84.8	82.4
Phan et al. [29]	79.1	69.8	0.70	75.5	27.3	86.0	85.6	74.8
Phan et al. [34]	79.8	72.0	0.72	77.0	33.3	86.8	86.3	76.4
Phan et al. [35]	81.9	73.8	0.74	-	-	-	-	-
Zhu et al. [36]	82.8	77.8	-	**90.3**	**47.1**	86.0	82.1	**83.2**
Yang et al. [27]	82.13	73.5	0.75	87.8	23.0	86.2	**90.9**	81.8
**CAttSleepNet**	**84.1**	**78.2**	**0.78**	89.6	**47.1**	**87.2**	85.0	82.1
**Dataset: Sleep-Edfx-2013 EEG Channel: Pz-Oz**								
Supratak et al. [20]	79.8	73.1	0.72	88.1	37	82.7	77.3	80.3
Yang et al. [27]	80.54	68.7	0.72	85.3	17.5	85.0	78.2	75.8
**CAttSleepNet**	**82.58**	**76.69**	**0.76**	**89.5**	**46.4**	**85.8**	**80.9**	**80.8**
**Dataset: Sleep-Edfx-2018 EEG Channel: Fpz-Cz**								
Mousavi et al. [37]	80.03	73.55	0.73	91.72	**44.05**	82.49	73.45	76.06
**CAttSleepNet**	**80.81**	**74.56**	**0.74**	**92.12**	43.74	**83.28**	**76.96**	**76.70**
**Dataset: Sleep-Edfx-2018 EEG Channel: Pz-Oz**								
Mousavi et al. [37]	77.56	70.00	**0.69**	-	-	-	-	-
**CAttSleepNet**	**78.01**	**70.08**	**0.69**	89.95	39.24	81.07	68.06	72.07

Note: The highest performance metrics are highlighted in bold. Except for the K indicator, values of other indicators are all percentiles.

**Table 5:** Ablation experiments on Fpz-CZ channel of the sleep-edfx-2013 dataset.

	CAttSleepNet (%)			CAttSleepNet without Attention (%)		
	Pre	Rec	F1	Pre	Rec	F1
W	88.86	90.28	89.56	83.13	83.13	83.14
N1	55.59	40.87	47.11	48.63	40.09	43.95
N2	86.24	88.21	87.22	90.05	88.20	89.57
N3	86.57	83.47	84.50	78.07	78.92	78.49
REM	80.05	84.30	82.12	75.58	87.64	81.87
Overall Indicators	**ACC**	**K**	**MF1**	**ACC**	**K**	**MF1**
	84.14	78.09	78.20	81.95	74.50	75.26

Note: Pre = precision, Rec = recall, F1 = F1-score.

## Data Availability

EEG signals used in our experiments are derived from the public Physionet sleep-edf expanded dataset.
